# COVID-19 Vaccination Coverage and Factors Influencing Vaccine Hesitancy among Patients with Inborn Errors of Immunity in Latvia: A Mixed-Methods Study

**DOI:** 10.3390/vaccines11111637

**Published:** 2023-10-25

**Authors:** Zane Lucane, Mirdza Kursite, Kristaps Sablinskis, Linda Gailite, Natalja Kurjane

**Affiliations:** 1 Department of Biology and Microbiology, Riga Stradins University, LV-1007 Riga, Latvia; 2 Department of Public Health and Epidemiology, Riga Stradiņš University, LV-1007 Riga, Latvia; 3 Department of Internal Diseases, Riga Stradins University, LV-1007 Riga, Latvia; 4 Scientific Laboratory of Molecular Genetics, Riga Stradins University, LV-1007 Riga, Latvia; 5 Outpatient Clinic, Pauls Stradins Clinical University Hospital, LV-1002 Riga, Latvia; 6 Outpatient Clinic, Children’s Clinical University Hospital, LV-1004 Riga, Latvia

**Keywords:** vaccine hesitancy, vaccination coverage, COVID-19 vaccine, SARS-CoV-2 vaccine, inborn errors of immunity, primary immunodeficiencies, Latvia

## Abstract

Background: The European Society for Immunodeficiencies recommends that all patients with inborn errors of immunity (IEI) without contraindications should receive SARS-CoV-2 vaccination. The aim of this study was to investigate the reasons that discourage IEI patients from receiving the recommended vaccination and to assess vaccination coverage among IEI patients in Latvia. Methods: In this multicenter mixed-methods study, the vaccination status of all patients with IEI within two tertiary centers in Latvia was reviewed using electronic health records. Semi-structured interviews were conducted with 16 IEI patients who did not undergo vaccination, and a thematic analysis was performed. Results: A total of 341 patients (49.3% female; median age 19.7 years (IQR:17)) were included in the quantitative part. The proportion of fully vaccinated individuals aged ≥ 12 years was 66.8%–70.9% with patients with selective IgA deficiency and 58.8% with other IEI (χ² = 14.12, *p* < 0.001). The proportion of fully vaccinated individuals aged 5–11 years was 11.1%. Age was associated with vaccination status: younger patients were found to have a significantly lower likelihood of receiving vaccination (U = 8585, *p* < 0.001). The five main themes identified were as follows: (1) fear and uncertainty; (2) risk and benefit assessment: COVID-19 vaccine—is it worth it? (3) external influences: the dark horse of the decision-making—people around us; (4) individuals against the system; and (5) beliefs about vaccination and COVID-19. Under-representation of certain IEI groups and recall bias are possible limitations of this study. Conclusions: While most reasons for hesitancy were similar to those previously described in the general population, disease-specific concerns were also identified.

## 1. Introduction

Inborn errors of immunity (IEI) encompass a diverse collection of inherited disorders characterized by defects in various parts of the immune system [1]. The defining characteristic of these disorders is immunodeficiency, which translates to increased susceptibility to infectious agents, with the specific causative agents varying depending on the particular diagnosis [2]. Although definitive treatment options, such as stem cell transplantation or gene therapy, for certain IEIs may be feasible, only supportive interventions such as lifelong immunoglobulin replacement therapy and biological or antimicrobial therapy are currently available for the majority of patients. Therefore, comprehensive management also requires a significant focus on infection prevention, where vaccination plays a pivotal role [3].

At the beginning of the coronavirus disease 2019 (COVID-19) pandemic, individuals with specific IEI were assumed to be more susceptible to severe acute respiratory syndrome coronavirus 2 (SARS-CoV-2) infection and experience more severe presentation compared to the general population [4,5,6]. Therefore, after vaccines became available, the European Society for Immunodeficiencies (ESID) recommended that all IEI patients devoid of contraindications should receive vaccination with any of the accessible vaccine formulations according to their national vaccination schedule to include primary courses and booster doses of vaccination, provided that they are not live vaccines for those in whom it would be contraindicated. Patients who were infected with SARS-CoV-2 and recovered were still recommended to be vaccinated against SARS-CoV-2 [7].

According to the World Health Organization’s (WHO’s) strategic advisory group of experts on immunization, vaccination hesitancy can be defined as a “delay in acceptance or refusal of vaccination despite availability of vaccination services” [8], and it was identified as one of the top ten global health threats already prior to the COVID-19 pandemic [9]. While vaccine hesitancy itself is a well-known phenomenon, it would be tempting to assume that patients who are aware of their defective immune system would automatically accept safe and recommended SARS-CoV-2 vaccines, since the assessment of the potential threat of COVID-19 is described as a motive for the intention to vaccinate [10]. However, an initial ambivalent attitude toward SARS-CoV-2 vaccination has been reported among some IEI patients due to limited data on vaccine efficacy and safety specific to this population [11], despite recommendations from specialists [7]. Nevertheless, when subsequent investigations were undertaken to assess the effectiveness and safety of vaccines specifically within this patient group, the reported safety data were reassuring [12,13,14]. Notably, although certain IEI groups have displayed diminished antibody responses against SARS-CoV-2 vaccination, the potential advantages of vaccination were deemed superior to the associated risks [15,16,17,18]. Furthermore, it is noteworthy to mention that T-cell immune responses in these patients remained largely unimpaired, highlighting the benefits of vaccination even if patients fail to mount a sufficient measurable antibody response [13,16,19,20,21,22].

Nevertheless, there have been cases in which patients declined vaccination or refrained from vaccinating their children against SARS-CoV-2, despite the inherent vulnerability of these individuals to various infectious diseases. Could the rationale underlying vaccine refusal in this patient population differ from those prevalent among the general population or patients with other chronic diseases? While several extensive studies have examined reasons for vaccination hesitancy among the general population [23,24,25] or other chronic diseases, such as autoimmune diseases [26,27,28,29,30,31,32,33] or cancer [34,35,36], data regarding reasons for the hesitancy of patients affected by inborn errors of immunity to accept vaccination against SARS-CoV-2 infection are limited. Few studies have addressed the reasons for vaccination hesitancy in this patient group, none of which had a qualitative design [11,37,38]. We hypothesized that employing a qualitative approach might uncover novel factors contributing to vaccination hesitancy, which may not have been previously documented in the general population or anticipated through researcher-designed multiple-choice queries and could be distinctive to individuals with IEI. Notably, a multinational study concluded that hesitancy to vaccinate was more prevalent in Eastern European countries than in Western Europe or North America [37]. The aim of this study was to investigate the reasons that discourage IEI patients from receiving the recommended SARS-CoV-2 vaccination and to assess vaccination coverage among IEI patients in Latvia.

## 2. Materials and Methods

### 2.1. Design

In this mixed-methods study, to assess vaccination coverage among patients with IEI and identify individuals who had not received vaccination against SARS-CoV-2, data regarding the SARS-CoV-2 vaccination status of all patients diagnosed with IEI in Latvia were extracted. Additionally, to gain insights into the reasons behind vaccination hesitancy, a qualitative approach employing thematic analysis of transcripts from semi-structured interviews was used. Qualitative methods in health care are commonly employed to gain deeper insights into complex psycho-social phenomena, rather than ‘measure’ them. It is widely regarded as the most suitable research approach for investigating human experiences, beliefs, and perceptions [39]. In this study, quantitative and qualitative data were analyzed separately and merged in the discussion.

### 2.2. Quantitative Study

#### 2.2.1. Study Population

Data were sourced from the only tertiary centers in Latvia, where immunologists consult (both children and adults): Children’s Clinical University Hospital and Pauls Stradins Clinical University Hospital. All patients diagnosed with IEI at these two facilities comprised the study population. Data were extracted from patient health records and included the following: age, gender, clinical diagnosis, treatment, and SARS-CoV-2 vaccination status. The diagnosis of IEI was based on the ESID diagnostic criteria [40]. Clinical diagnoses were classified according to the International Union of Immunological Societies (IUIS) classification [2].

#### 2.2.2. Statistical Analysis

Data related to demographic and clinical indicators were analyzed using descriptive statistics and parametric/non-parametric analysis, as appropriate. The Shapiro–Wilk test was used to determine whether continuous variables were normally distributed. The results indicated that the age data were not normally distributed; therefore, medians and interquartile ranges (IQRs) were used in data presentation, and nonparametric statistical methods were used in subsequent analyses. The differences in categorical variables were examined using the Chi-square and Fisher exact tests. The Mann–Whitney U was used to compare continuous variables between two groups. Statistical significance was set at *p* value < 0.05. Statistical analysis was performed using IBM SPSS Statistics version 29 (IBM, New York, NY, USA).

### 2.3. Qualitative Study

#### 2.3.1. Study Population and Sample

A purposive maximum variation sampling strategy [41] was used to recruit patients, representing diverse demographic parameters (age group and gender) and a variety of diagnostic groups according to the IUIS classification. The sample consisted of both adult patients with IEI and parents of children with IEI who were eligible for vaccination, and none of the patients had received any SARS-CoV-2 vaccine.

#### 2.3.2. Data Collection

Semi-structured interviews were used for qualitative data collection. The one-on-one semi-structured interview method allows the researcher to set an agenda in terms of the topics covered, while also allowing participants the freedom to express themselves openly. Therefore, it represents the optimal method for gathering data on individuals’ perspectives, beliefs, and perceptions regarding sensitive topics [39]. The semi-structured interview protocols (see Supplementation S1) were designed following a literature review and were grounded in the Health Belief Model (HBM), a theoretical model that attempts to explain why certain health behaviors are adopted, such as preventive health and vaccine use [42]. The HBM contains six constructs: encompassed perceived susceptibility (assessing the risk of becoming ill), perceived severity (evaluating the seriousness of the illness or unwanted potential consequences), perceived barriers (analyzing the influences that discourage adoption of vaccination), perceived benefits (appraising the positive outcomes associated with vaccination), cues to action (events, people, or things that trigger the behavior change), and self-efficacy (confidence and belief in the ability to perform a behavior) [42]. The main emphasis was on the “barriers” construct, and questions exploring the “self-efficacy” construct were not included, since we did not predict that this issue would be the driving force that would retrain immunodeficient patients from vaccination, as they were not reported by previous studies in IEI patients [11,37,38]. Additionally, the input was gleaned from validated vaccine hesitancy evaluation instruments and explanatory models [43,44,45,46,47,48], as well as insights gained from a pilot interview. Interview questions were offered to participants in two languages, Latvian and Russian, to facilitate effective communication.

Eligible patients were contacted via telephone, and upon obtaining their consent to participate in the study, the interviews were conducted via telephone call. With the participants’ consent, the interviews were audio-recorded and transcribed verbatim for analysis. After the transcription of the interviews, each patient was assigned a code that was used throughout the study to guarantee anonymity. Interviews were conducted from April to August 2023 by one member (Z.L.) of the authoring team at a time chosen by each participant and lasted from 7 to 55 minutes (average time—20 minutes). The interviews with these patients continued until a point of data saturation was reached, signifying that no new unique themes emerged during the final two interviews.

#### 2.3.3. Data Analysis

The interview transcripts were analyzed using NVivo plus version 12 (Lumivero, Denver, CO, USA). A thematic inductive analysis with ‘open coding’ was performed on the transcripts of the interview data. The basis of thematic analysis is to reduce the complexity of the data by looking for patterns or “themes” in the data [39,49,50]. In the thematic analysis, six phases according to Braun and Clarke [49] were adopted: (1) familiarization with the data, (2) generating initial codes, (3) searching for themes, (4) reviewing themes, (5) defining and naming themes, and (6) producing the report. In the process of thematic analysis, we scrutinized the coded data and observed that certain codes exhibited a pattern, and these codes were systematically grouped into overarching themes that conveyed insights relevant to the research question. The initial themes were then reviewed, modified, and refined to “identify the ‘essence’ of what each theme is about” [49]. Data analysis was performed independently by two members of the authoring team, Z.L. and K.S. Both authors coded four interviews independently and then met to discuss the initial codes, overarching themes, and disparities in analysis, which were discussed until a consensus was achieved. To ensure the rigor and accuracy of the analysis, the researchers were consulted by an experienced researcher with a background in qualitative research. The authoring team engaged in a collaborative process to review and enhance the identified themes and subthemes.

#### 2.3.4. Ethics Statement

This study was conducted in accordance with the principles of the Declaration of Helsinki. The study protocol was part of a study reviewed and approved by the Central Board of the Ethics Committee of the Health Ministry of the Republic of Latvia (No. 01–29.1/2878). All participants read and understood the informed consent that included information about the study, including its purpose, procedures, potential risks, and benefits, before agreeing to participate in the study; they also had an opportunity to ask any questions, which were answered by our team members. All participants provided a written form of consent prior to the interviews. They were assured of their complete anonymity and confidentiality. To safeguard their privacy, all identifiable information was carefully removed. Additionally, all data collected in this research were securely stored and accessible only to the research team. Participants were also informed that participation was completely voluntary and that they had the right to withdraw from the study at any point without facing any negative consequences.

This study adhered to the Standards for Reporting Qualitative Research guidelines (COREQ) [51].

## 3. Results

### 3.1. Quantitative Findings

#### 3.1.1. Population Characterization

In the present study, a cohort of 341 patients was analyzed to assess vaccination rates among individuals with IEI. The median age was 19.7 years (IQR: 17; range: 0–78 years), 49.3% were female, and 44.2% were pediatric patients (see Table 1). Most of the patients living with IEI were diagnosed with predominantly antibody deficiency (64.2%), followed by combined immunodeficiency with syndromic features (17.6%). The majority of the patients (57.2%) were diagnosed with selective IgA deficiency (SIgAD). In the SIgAD group, the median age was 22 years (IQR: 15 years; range: 5–78 years), 43.1% were female, and 31.3% were pediatric patients. In the group of 146 patients with IEI other than SIgAD, the median age was 15.1 years (IQR: 19.5; range: 0–72 years), 57.5% were female, and 61.5% were pediatric patients.

#### 3.1.2. Vaccination Coverage

Of all patients with an IEI ≥ 12 years and for whom information regarding vaccination status was available (n = 238), 66.8% were vaccinated (see Table 2). In the age group of 5–11 years, of the 54 patients for whom the information was available, 11.1% had received the vaccination, while in the age group of 6 months to 5 years none of the patients had received the vaccination. In the patient group aged ≥ 12 years, age was significantly associated with vaccination status, and younger patients were found to have a significantly lower likelihood of receiving vaccination (U = 8585, *p* < 0.001). This association between vaccination status and age was not observed if analysis was conducted in the adult group separately. No association was found between the vaccination status and gender.

Data from SIgAD patients and patients with more severe IEI were also analyzed separately because of the specific characteristics and milder phenotype of SIgAD that distinguish it from other types of IEIs. The results of the Chi-square test revealed a significant association between diagnosis (SIgAD or other IEI) and vaccination status (χ² = 14.12, *p* < 0.001). Cramer’s V indicated a small effect size (V = 0.21) for this association.

Of the SIgAD patients ≥ 12 years of age and for whom information regarding vaccination status was available (n = 158), 70.9% were vaccinated, while in the age group of 5–11 years (n = 19), this number was 15.8%.

Of all other IEI patients aged ≥ 12 years and for whom vaccination status was available (n = 80), 58.8% had received SARS-CoV-2 vaccination, while in the age group of 5–11 years (n = 35), this number was 8.6%. After controlling for age, vaccination status was not found to be associated with immunoglobulin replacement therapy.

### 3.2. Qualitative Study

#### 3.2.1. Study Sample Characterization

Seventeen patients were invited to participate in the interviews, and one refused. Finally, sixteen participants were included in the qualitative study (see Table 3); nine of these were parents of children with IEI, whereas six were adult patients with IEI. Six patients were male and ten female. Four of sixteen patients were receiving immunoglobulin replacement therapy.

#### 3.2.2. Findings of Thematic Analysis

Inductive thematic analysis revealed five themes and twelve subthemes for reasons that increment vaccination hesitancy or refusal (see Table 4). Identified themes (5) and subthemes (12) are described below, with participant quotations translated into English and identified by participant code. For the majority of patients, vaccination hesitancy was influenced by a combination of interconnected and mutually reinforcing reasons. Most patients reported fear and a higher perception of risks associated with vaccination (health concerns) and/or a low perception of benefits (possibly impaired immune response to vaccination) that drove them toward hesitant attitudes about vaccination. This risk/benefit evaluation was influenced by several external and internal factors, such as influences from other people’s opinions, mistrust, personal beliefs about vaccination and COVID-19, and negative personal experiences.

### 3.3. Theme 1. Fear and Uncertainty

Fear and uncertainty appeared to play an important role in shaping participants’ decision-making about the SARS-CoV-2 vaccination, with some individuals citing these factors as the primary reasons for their refusal of the vaccine.

#### 3.3.1. Fear

Notably, fear as a key emotion was mentioned by many participants. Mainly, participants expressed unspecified fear of vaccination.

“But even now I would not like to vaccinate him, because I am afraid, because I am afraid of some kind of reaction and that something will be bad.” (IEI2)

Among those who could identify the source of their fear, predominant factors included health concerns, as well as a fear of death.

“Fear of dying. I want to live a little longer.” (IEI14)

#### 3.3.2. Too Many Unknowns

Uncertainty regarding the overarching pandemic context emerged as a recurrent theme within interviews. While not necessarily mentioned as a direct reason for vaccine refusal, it appeared as a closely linked theme alongside vaccine hesitancy. Some participants emphasized the feeling of confusion, not knowing what to consider to be true and what a myth.

“All of those horror stories somehow influence the psyche.. yes.. because the contrary information is not that much. Where is the truth—no one really knows.” (IEI5)

### 3.4. Theme 2. Risk and Benefit Assessment: COVID-19 Vaccine—Is It Worth It?

A diminished perception of possible benefits in combination with intense health-related concerns led certain participants to perceive the risks associated with vaccination as outweighing its potential benefits.

“Well, yes, there are advantages, but there are more disadvantages than advantages.” (IEI14)

#### 3.4.1. Concerns about Negative Outcomes

The majority of participants expressed some concern regarding vaccination-associated health issues, encompassing both known adverse effects and potential long-term consequences that may still remain uncertain.

“All those listed side effects.. For example, I feel like I will get them all at once. Perhaps, it is better not to get vaccinated. For example, a blood clot formation [can occur]. I simply believe that I don’t need it.” (IEI12)

Concerns regarding the possibility of disease exacerbation following SARS-CoV-2 vaccination appeared to be a recurrent theme among participants.

“Well, in general, it could be that things go smoothly, but maybe something with the immunity goes wrong again, not as it should be.” (IEI8)

#### 3.4.2. Doubts about the Positive Effect

Participants, mainly in the predominantly antibody deficiency diagnosis group, had low expectations that there would be a desired effect after vaccination since they were informed that antibody responses following vaccination may be impaired.

“Well, one thing is that I may not have the antibodies after vaccination anyway.” (IEI14)

Another reemerging theme was doubting the necessity of vaccination because a vaccinated person can also get infected. Several participants mentioned stories from their immediate social circles where unvaccinated people did not get infected or had milder symptoms of COVID-19 than vaccinated people.

“I had a wedding, we were 10 people, and 5 of them got sick. Of those who became sick—3 them were very ill, and all three were vaccinated three times. The other two, who were not vaccinated, were me and my husband. We did not feel anything. And we all just got tested, and it turned out that half got sick and half didn’t, and even those who were not vaccinated didn’t get sick.” (IEI10)

#### 3.4.3. Overall Lack of Confidence in the Vaccine

Several participants expressed concerns and skepticism about the science behind the SARS-CoV-2 vaccination. They emphasized that SARS-CoV-2 vaccines are under-studied and too novel to be used, particularly for patients with immune system diseases. Even though some participants displayed a general lack of confidence in all vaccines, the prevailing attitude among most of the participants was skepticism, which was focused primarily on SARS-CoV-2 vaccination.

“Well, there are vaccinations where you have to see what the vaccine is, because untested vaccines and vaccines that have just started to be used, if they have not yet evolved over time, they are difficult for people like me.” (IEI15)

### 3.5. Theme 3. External Influences: The Dark Horse of the Decision-Making—People around Us

As one of the major themes, the opinions and stories of other people were mentioned as an important factor in making the decision about vaccinating against SARS-CoV-2.

#### 3.5.1. Stories of Health Problems after Vaccination

Numerous participants recalled stories about different health problems that appeared after vaccination against SARS-CoV-2. Some participants shared stories from family members or close friends and others from distant sources. All of them, with various degrees of certainty, expressed the opinion that negative health outcomes were due to vaccination.

“My daughter’s best friend’s stepfather died within a month [after vaccination], he developed stomach cancer, which was not diagnosed before, right? I’m surrounded by [cases similar to these]—if you think about it, I could write a book here.” (IEI15)

“And then my husband got one vaccine, and that’s it. After that, he had a gout flare approximately two weeks later. It was something that he had experienced only once in the past, and now it’s happening very frequently to him, for instance.” (IEI10)

#### 3.5.2. Public Figures and Doctors against Vaccination

Several participants noted that hearing public figures and doctors being against vaccination made them doubt it as well. Most commonly, polarized opinions among the medical community were mentioned. For some participants, doubts about the necessity of the SARS-CoV-2 vaccination were influenced by their attending physician or other specialists.

“And also, as government officials expressed: “I didn’t vaccinate my daughter because then she might be at risk of not having children.” (IEI3)

“One person says one thing, another says another, and it’s the same with the COVID vaccination—some say it’s not necessary, even a couple of doctors told me it’s better not to risk, while others say it’s definitely needed, and it seems that no one really knows for sure.” (IEI10)

### 3.6. Theme 4. Individuals against the System

Another major theme was a collision of individual thoughts and wishes and the narrative of governmental and healthcare institutions.

#### 3.6.1. The Right to Choose

Numerous participants emphasized the feeling that the vaccination was forced on them, leaving them with a sense of their autonomy regarding their bodily choices being constrained.

“I believe that everyone is responsible for their own body. Everyone has a choice of whether to get vaccinated or not. But when they imposed all this foolish coercion, it further diminished any trust in the whole [vaccination process].” (IEI12)

“I am precautionary of the fact that, let’s say, I had a colleague of mine, she had an oncological disease, by the way, she did not cope with the disease [COVID-19], but she was an anti-vaccination. Yes, she died in the end, yes, but at least she stayed with her own opinion.” (IEI16)

Some reported that the compulsory vaccination campaign created more resistance to the SARS-CoV-2 vaccination and that they would have considered it if it had not been mandatory.

“I don’t think people need to be forced. Here is the vaccine to do for those who want to do it, who does not want to do it should not be forced. So I think that this is what they can say so they forced it wrong. They made more resistance with this, maybe I would even go if they didn’t force me so much.” (IEI2)

#### 3.6.2. Mistrust

Mistrust emerged as a prominent theme in the interviews and was manifested at different levels. Some participants expressed skepticism about the interests behind vaccines and about the vaccine developers and pharmaceutical companies. Notably, this sense of distrust extended to the healthcare system and governmental institutions, with mutual influence observed among different levels and types of mistrust.

“[I obtain the information] from the Disease Prevention and Control Centre, etc. [The information] from the government—I don’t trust it.” (IEI5)

“Most likely, us, ordinary people, do not have a deep understanding—is it really as the doctors say?” (IEI6)

#### 3.6.3. General Disbelief of Official Information

Some participants expressed disbelief and doubts about the truthfulness of publicly available information about COVID-19, vaccination against it, or vaccination in general. They mentioned the pandemic and vaccination being a fraud.

“[laughs] Well, I think it was not even a real illness. Just some kind of virus. Everyone just said COVID, COVID.” (IEI11)

“Now I am starting to read some publications on the Internet, that it was some kind of scam with these COVID vaccinations. They write that they are now revealing with these pharmaceutical companies and governments, they are involved in a scam” (IEI7)

### 3.7. Theme 5. Beliefs about Vaccines and COVID-19

Throughout interviews, different types of beliefs about vaccines or COVID-19 emerged as one of the contributing factors in the decision-making process of whether to get vaccinated both in general and particularly against SARS-CoV-2.

#### 3.7.1. Beliefs about Vaccination in General

Different beliefs related to vaccination emerged in the interviews that were stated as some of the reasons for their unwillingness to vaccinate. Some participants mentioned that vaccines “weaken” or “overstimulate” the immune system, while others mentioned that vaccination is not necessary for treatable diseases, or might cause autism.

“My son, too, had whooping cough after being vaccinated. At the age of 14 he had whooping cough. He was treated, we managed without a hospital, but there was a strong cough, there was a very strong cough. I think that it is not necessary to be vaccinated against it, since it can be treated with medication, and those diseases that are not treatable, it seems to me that maybe it’s worth getting vaccinated.” (IEI7)

Also, disease-specific beliefs were mentioned, such as that vaccinations are contraindicated for rare diseases or that previous vaccinations may have been the cause of immunodeficiency itself. Some participants shared the belief that receiving immunoglobulin replacement therapy was sufficient for immunity and, therefore, did not need the vaccine.

“First and foremost, I have children for whom vaccination is not allowed at all. They have rare diseases, and they must not receive anything like that at all.” (IEI11)

“Well, somehow it seems to me that vaccines are not good for him. I think they may be dangerous. And plus, if immunoglobulins give him everything he needs for protection, then why would he need it?” (IEI2)

#### 3.7.2. Beliefs about COVID-19 or the Vaccine against It

Some participants expressed the belief that COVID-19 was not a severe disease, also mentioning examples of immunodeficient people who had mild or no symptoms.

“When my child got sick with COVID for the first time, I thought I would lose him, but during all 3 times when he was sick, he didn’t even have a fever. How can you have such a disease, if you don’t even have lungs to breathe, where the smallest thing could hurt you, and you get COVID, but you have nothing [from the clinical symptoms]?” (IEI4)

Other participants talked about different reasons why they thought that the vaccination was not necessary in their particular situation, for example, after being infected with COVID-19 or substituting vaccination with isolation from others.

“But she hasn’t been vaccinated because we didn’t make it on time, and now it doesn’t make sense. Because she was sick all the time. That period when I thought, well, let’s do it, then she got sick for the second time—she was infected with COVID without vaccination, so we thought we would wait for that period, that we didn’t need to vaccinate yet. And then there was the second episode. But this time, of course, in much milder form.” (IEI6)

## 4. Discussion

This study is the first to employ qualitative interviews to gain a more comprehensive understanding of the motivations underlying IEI patients’ decisions to decline vaccination, as well as access to vaccination coverage among IEI patients in Latvia. In this study, the proportion of fully primary-vaccinated individuals above 12 years of age was 66.8%, which is similar to that in the Latvian general population (68%) [52]. The main themes identified behind the vaccination hesitancy were as follows: (1) fear and uncertainty, (2) risk and benefit assessment: COVID-19 vaccine—is it worth it? (3) external influences: the dark horse of the decision-making—people around us, (4) individuals against the system, and (5) beliefs about the vaccination and COVID-19.

Previous studies in the general population have shown that sociodemographic factors, such as gender, education level, race, and ethnicity, are the most prominent factors associated with the intention to use SARS-CoV-2 vaccines [25,44]. Concerning demographic factors within the IEI group, an extensive cross-national analysis involving 40 countries revealed a linkage between female gender and greater vaccine hesitancy [37], while findings from a study conducted in Poland indicated that unvaccinated individuals were more likely to possess primary or vocational education [38]. In this study, unlike in previously mentioned IEI cohorts, age was associated with vaccination status; however, this difference can be ascribed to the predominance of pediatric cases within our cohort, where the median age was 19.7 years, contrasting with the median ages of 42–47 years in other cohorts [37,38].

While the overall vaccination rate was comparable to that in the general population, after excluding SIgAD patients, vaccine coverage in the IEI group other than SIgAD patients above 12 years of age was 58.8%, which is lower than that in the general population in Latvia. This contrasts with other studies, where the reported vaccine acceptance rate was higher in IEI patients compared to the general population [11,37,38], while the share of SIgAD patients in these cohorts was much lower (0.006%–6.9% of all included patients [37,38] vs. 57.8% in our study group). Furthermore, the qualitative analysis revealed that, in addition to universal vaccine refusal reasons, such as concerns about adverse effects, patients with IEI also had disease-specific concerns. This may have contributed to a heightened perception of vaccination as a threat, as compared to individuals in the general population. Interestingly, another study in the Latvian general population revealed that perceived vulnerability was not identified as a factor related to SARS-CoV-2 vaccination [45], unlike in other countries [24]. This is consistent with our finding that the vaccination rate in patients with the most vulnerable immune system was the lowest.

Previous studies in patients with IEI have reported uncertainty regarding whether an immune response can be mounted as the most common reason. This primary concern was then followed by concerns about allergic reactions, still unknown vaccine side effects, or post-vaccination disease flares [11,37]. Both the identified themes, namely, concern about vaccine efficacy given the immunodeficiency state, as well as concern about negative health outcomes, were also represented in our study group. In addition, although fear was a frequently mentioned theme in our study group, participants mainly expressed fear of the SARS-CoV-2 vaccination and not the virus, although the majority of patients considered COVID-19 to be a severe illness. This prioritization of fear over vaccine-related risks compared to the fear of COVID-19 has been reported previously in the general population [53], and it can be understood through the HBM constructs, including perceived susceptibility, severity, benefits, and barriers [42].

Several external and internal influences that shape IEI patient attitudes and behaviors have been described in previous studies, including advice from healthcare providers, skeptical viewpoints of the science behind the SARS-CoV-2 vaccine, and mistrust in the medical system in general [11]. In previous studies, for most patients, the concerns were SARS-CoV-2 vaccine-specific, while only 1.7–4.7% expressed mistrust in vaccines in general [11,37]. Themes such as external influence by their healthcare provider or other authorities, mistrust, as well as false beliefs were also reported by our participants. While some false beliefs were commonly reported vaccination myths, such as that vaccines might “overwhelm” or “weaken” the immune system [54], others seemed to be specific to the IEI population, such as a belief that vaccination is not necessary if the patient receives immunoglobulin replacement treatment. This belief could potentially stem from a misinterpretation of the information provided by an immunologist since this belief is true for other vaccine-regulated diseases that have sufficient vaccine coverage in the general population for which these preparations provide passive immunity [55]. However, at the beginning of the COVID-19 pandemic, this belief did not adhere to current recommendations [7]. First, immunoglobulin product preparation is a complicated process that takes several months; therefore, a delay in the appearance of sufficient levels of SARS-CoV-2 specific antibodies in these products is expected, which was especially important at the beginning of vaccination campaigns [56]. Second, even if a particular patient fails to mount a specific antibody response to vaccination, T-cell immune responses may be intact and serve as an additional defense against SARS-CoV-2 infection [57,58,59,60]. In addition, the anticipation of vaccines offering complete sterile immunity, coupled with a tendency to assess vaccine efficacy not through empirical evidence but rather through a limited number of cases observed within one’s immediate social circle, constituted an additional factor contributing to participants’ skepticism towards vaccine efficacy. These misconceptions may be associated with inadequate levels of health literacy within the Latvian population. A recent investigation indicated that approximately 79% of Latvian citizens exhibit weak health literacy competencies, potentially contributing to riskier health-related behaviors, such as vaccine hesitancy [61].

Narratives regarding the negative health consequences of the SARS-CoV-2 vaccination have been circulating around and have been reported by studies in the Latvian general population [45], as well as in our patients, including death purportedly linked to SARS-CoV-2 vaccination. In some cases, these perceptions were grounded in unsubstantiated convictions that there is a direct causal link between SARS-CoV-2 vaccination and deteriorating health conditions. Several of our participants, those who expressed concerns about negative vaccination health effects, also admitted that they had gained this information from social media platforms. Indeed, social media discourse in countries that focus more on side effects or negative emotions is correlated with a lower country’s vaccination rate [62]. In addition, vaccine skeptics frequently use online platforms to spread disinformation about vaccines [63].

Interestingly, another study in the Latvian general population revealed that, among others, institutional trust and fear of COVID-19 are predictors for SARS-CoV-2 vaccination behaviors [64]. Trust in the context of vaccination can be conceptualized across three distinct dimensions: confidence in the vaccine product itself (e.g., SARS-CoV-2 vaccine), reliance on the vaccine provider (e.g., medical professionals), and faith in policymakers (e.g., the government and the healthcare system) [65]. According to our data, patients’ perception of vaccine efficacy and safety seemed to be influenced by mistrust in all three of these levels. Interestingly, in previous studies among the general population, education was reported to have less explanatory power of vaccine hesitancy, suggesting that a deficit in scientific literacy may play a lesser role than the establishment of public trust in the health system and science [66]. Indeed, mistrust in state institutions has been reported to directly affect the population’s willingness to act in accordance with the recommendations of these institutions, while trust and confidence in the state and healthcare system has been associated with greater compliance to SARS-CoV-2 vaccination in Europe [67]. Social and economic inequality has also been associated with lower SARS-CoV-2 vaccination coverage [68], as well as higher indicators of public corruption, which is believed to generate mistrust in government [69,70]. Indeed, according to the Eurobarameter, trust in political parties as well as healthcare professionals is among the lowest in the EU [71] and corruption tolerance [72], as well as the Gini coefficient, are higher than average in the EU [73], which is also reflected in data regarding vaccination hesitancy in Latvia [45]. Mistrust in policymakers is also thought to be an important aspect of formation of conspiracy beliefs, together with psychological and structural factors [74,75,76].

Altogether, these findings underscore the importance of providing training to physicians and nurses regarding the effectiveness and safety of SARS-CoV-2 vaccination in individuals with IEI [37]. Trust between doctors and patients is particularly important for vaccination decisions [77]. In addition, some patients expressed that they feel unique with this condition; therefore, generic information by public health professionals may not seem fully applicable to them due to their specific condition. Therefore, from the perspective of vaccine communication, healthcare professionals would play a pivotal role in building trust, communicating about the personal benefits of vaccination, and addressing misinformation and hesitancy concerns regarding SARS-CoV-2 vaccines for these specific patients with a rare disease [77]. It has been shown that healthcare professionals’ recommendation to receive the vaccine has a positive effect on vaccination behavior, while the lack of confidence because the vaccine was too new showed a negative effect on vaccination intention [78]. In addition to that, initiatives that increase health literacy could be employed, including health education in schools and the provision of structured health-related information about vaccinations [61].

This study has several limitations. First, the data were gathered between April and August 2023, a period following the end of the pandemic in May 2023. Given that vaccination initiatives were predominantly conducted earlier, the potential for recall bias exists, which may lead to missing details or the inclusion of inaccurate information in participants’ responses, leading to a misunderstanding of the topic. However, this time framework also enabled the exclusion of interviewees who were inclined towards vaccination, as those who harbored such intentions had enough time to proceed with vaccination. Second, there is a likelihood of under-representation of specific IEI classification categories, particularly patients with congenital neutropenia or bone marrow failure treated by hematologists in Latvia. This could potentially result in the omission of diagnoses that may have disease-specific concerns due to their unique clinical characteristics or treatment regimens (including HSCT), leading to an incomplete understanding of the issue and limited generalizability to all IEI patients. Furthermore, we used the HBM to design interview questions. However, several frameworks that could explain hesitancy have been developed, such as the 5C model of the drivers of vaccine hesitancy, which consists of five components: confidence, complacency, constraints, risk calculation, and collective responsibility [79]. In our interview questions, we did not include specific questions to explore constraints (availability or affordability), nor did we questions about confidence (or self-efficacy) and collective reasonability issues, since we did not predict that these issues would be the driving forces that would prevent immunodeficient patients from vaccination, as they were not reported by previous studies [11,37,38]. Notably, vaccination was readily accessible through dedicated vaccination centers or local general practitioner (GP) facilities in Latvia, with no associated costs, thereby minimizing concerns about accessibility or inconvenience as substantial influential factors. In addition, some possible associations were beyond the scope of this study, as we did not incorporate information concerning the educational level of the patients or other plausible factors that have been documented as influential in vaccination decisions, and their absence might lead to an incomplete picture of the issue. Furthermore, we did not use validated vaccination hesitancy scales due to the absence of validation in the Latvian language. However, the application of such scales within this particular population could be potential fields for future research. Employing standardized scales could reduce interviewer bias and facilitate comparative analysis with patients in other populations. Moreover, the incorporation of additional factors could enhance the comprehensiveness of the insights into vaccination hesitancy.

## 5. Conclusions

The reasons for SARS-CoV-2 vaccine acceptance and hesitancy in the IEI patient group in Latvia are complex and multifaceted, and both specific immunity status and the geopolitical context play a role. While most reasons for vaccination hesitancy in this group are similar to those in the general population, the findings suggest that individuals with immunodeficiency have unique perceptions and concerns that influence their decisions regarding vaccination. It is important to consider these factors when consulting IEI patients about vaccination.

## Figures and Tables

**Table 1:** Demographic characteristics, treatment, and vaccination coverage in different IEI categories according to IUIS classification.

IEI Category	Total Number of Alive IEI Patients	Age Group Pediatric/ Adult	Receives Immunoglobulin Replacement Yes/No	Vaccination Status Yes/No/ Non-Eligible for Vaccination */ No Information
Immunodeficiencies affecting cellular and humoral immunity	4	4/0	2/2	0/2/2/0
Combined immunodeficiencies with associated or syndromic features	60	53/7	2/58	11/33/16/0
Predominantly antibody deficiency	219	65/154	21/198	131/70/0/18
Of those, SIgAD	195	61/134	0/195	115/62/0/18
Diseases of immune dysregulation	4	3/1	2/2	1/3/0/0
Congenital defects of phagocyte number or function	18	10/8	0/18	5/10/1/2
Defects in intrinsic and innate immunity	4	3/1	2/2	2/0/2/0
Autoinflammatory disorders	12	9/3	0/12	4/6/0/2
Complement deficiencies	11	1/10	0/11	6/5/0/0
Bone marrow failure	6	1/5	0/6	5/0/1/0
Phenocopies of inborn errors of immunity	1	0/1	1/0	1/0/0/0
Unclassified immunodeficiency	2	2/0	0/2	0/1/0/1
Total	341	151/190	30/311	166/149/3/23

* Patients younger than 6 months old or SCID patients with elective hematopoietic stem cell transplantation.

**Table 2:** Vaccination coverage comparison between the Latvian general population, SIgAD, and other IEI patients in different age groups.

Age Group, Years	Latvian General Population, % Vaccinated [52]	Selective IgA Deficiency Patients, % Vaccinated	Other IEI Patients, % Vaccinated
12–19	68	51.0	26.5
20–29	81	82.8	72.2
30–39	77	80.0	90.9
40–49	79	70.0	60.0
50–59	81	80.0	83.3
60+	71	60.0	75.0
Total	68%	70.9%	58.8%

**Table 3:** Clinical and demographic characteristics of interview participants.

Code	Interviewee	Diagnosis Group	Receives Immunoglobulin Replacement	Gender of the Patient	Age Group of the Patient, Years
IEI1	Parent	Combined immunodeficiencies with associated or syndromic Features	No	F	5–11
IEI2	Parent	Predominantly antibody Deficiency	Yes	M	5–11
IEI3	Parent	Predominantly antibody Deficiency	No	M	5–11
IEI4	Parent	Diseases of immune Dysregulation	Yes	M	12–18
IEI5	Parent	Predominantly antibody Deficiency	Yes	M	5–11
IEI6	Parent	Predominantly antibody Deficiency	No	F	12–18
IEI7	Patient	Predominantly antibody Deficiency	Yes	F	40–60
IEI8	Parent	Combined immunodeficiencies	No	F	12–18
IEI9	Patient	Predominantly antibody Deficiency	No	F	>60
IEI10	Patient	Complement deficiency	No	F	19–40
IEI11	Parent	Combined immunodeficiencies with associated or syndromic Features	No	F	5–11
IEI12	Patient	Predominantly antibody Deficiency	No	F	19–40
IEI13	Parent	Autoinflammatory disease	No	F	12–18
IEI14	Patient	Predominantly antibody Deficiency	No	M	40–60
IEI15	Patient	Complement deficiency	No	F	40–60
IEI16	Parent	Combined immunodeficiencies with associated or syndromic Features	No	M	12–18

**Table 4:** Themes and subthemes of factors that influence vaccination hesitancy among IEI patients in Latvia.

Themes	Subthemes
1. Fear and uncertainty	Fear Too many unknowns
2. Risk and benefit assessment: COVID-19 vaccine—is it worth it?	Concerns about negative outcomes Doubts about the positive effect Overall lack of confidence in the vaccine
3. External influences: The dark horse of the decision-making—people around us	Stories of health problems after vaccination Public figures and doctors against vaccination
4. Individuals against the system	The right to choose Mistrust General disbelief of official information
5. Beliefs about vaccines and COVID-19	Beliefs about vaccines in general Beliefs about COVID-19 and its vaccine

## Data Availability

The datasets generated during and/or analyzed during the current study are available from the corresponding authors upon reasonable request.

## Supplementary Materials

The following supporting information can be downloaded at: https://www.mdpi.com/article/10.3390/vaccines11111637/s1. Supplementary material File S1: Interview questions.
