# Supplementary material for: COVID-19 Vaccination Coverage and Factors Influencing Vaccine Hesitancy among Patients with Inborn Errors of Immunity in Latvia: A Mixed-Methods Study
Source: Vaccines (Basel). 2023 Oct 25;11(11):1637. doi: 10.3390/vaccines11111637 (PMC10675738; doi:10.3390/vaccines11111637)
Supplement: Supplementary file 1 [file vaccines-11-01637-s001.zip › vaccines-2667949-supplementary.pdf]

Supplementation 1. Interview protocol

I Exploring personal beliefs and experiences about vaccination (barriers to vaccination)

1. Can you tell us about your views on vaccination, both COVID-19 vaccine and in general? (Additional: What prevents you from vaccination/vaccinating your children?)
2. Can you describe a personal experience that has influenced your decision to vaccinate or not to vaccinate/to vaccinate or not to vaccinate your child?
3. Have you been vaccinated/vaccinated your child against COVID-19 and infections other than COVID-19?

II Exploring external influences - from other people, religion, society, information sources

4. To what extent have other people's comments influenced your thoughts about vaccination? (Additional: Can you give an example?)
5. What role do religious beliefs play in your decision-making about vaccination? (Additional: Can you give an example?)
6. Do you feel any societal pressures that may influence your decision to vaccinate or not to vaccinate? (Additional: Can you give an example?)
7. How do you get information about vaccines and what sources do you trust? (Additional question: To what extent do you trust sources of information about vaccines, such as the government, healthcare providers and the media?)

III Exploring perceived disease severity, susceptibility, vaccination risks (barriers) and benefits

8. Speaking specifically about COVID-19, do you think it is a serious disease and are you/your child susceptible towards COVID-19?
9. Can you tell us about any concerns or fears you may have about the safety or effectiveness of vaccines?
10. What do you think are the benefits of vaccination?

IV Assessment of the communication of the responsible organisations

11. How do you feel about the recommendations and policies of government or health organisations on COVID-19 vaccination?
